# A rare case of scrotal emergency: torsion of epididymal cyst—a case report and literature review

**DOI:** 10.3389/fped.2023.1245842

**Published:** 2024-01-09

**Authors:** Yu Wang, Kai Wang, Longdi Yu, Xue Ma

**Affiliations:** Department of Pediatric Surgery, West China Hospital, Sichuan University, Chengdu, Sichuan, China

**Keywords:** epididymal cyst, torsion, scrotum emergency, ultrasonic examination, conservative treatment, scrotal exploration

## Abstract

As a benign disease, the incidence of epididymal cyst (EC) is 5%–25%, and it is relatively common in adults but rare in children. Although the pathogenesis of EC is still unclear, the symptoms of most asymptomatic patients can disappear with conservative treatment. EC torsion is even rarer in adults and children. Only ten cases have been reported in the literature, and we add this 3-year-old boy as the eleventh case of EC torsion. He was admitted to the hospital due to scrotum emergency without a history of EC or recent scrotal trauma. The ultrasonic examination revealed signs of an epididymal cyst. However, the pain and swelling of his scrotum were very similar to those of patients with testicular torsion, so we performed surgery on him. Therefore, EC torsion is very necessary to attract our attention as a differential diagnosis of testicular torsion in scrotal emergencies. Furthermore, our case is also the second youngest case of EC torsion.

## Introduction

EC is a cystic accumulation of fluid in the scrotum, which is generally considered to be benign and is usually characterized by painless swelling at the top of the testis. It is reported that the incidence is 5%–25%. ECs are thought to be relatively common in adults and rare in children, but they are more common before puberty than previously thought. At present, there is no consensus on the pathogenesis of EC, but it is generally considered to be acquired rather than congenital (1). EC can be easily diagnosed by scrotal ultrasound. The degree of scrotal swelling and whether it is accompanied by pain are usually the reasons for patients seeking medical attention. In most patients with epididymal cyst, the symptoms will disappear spontaneously after conservative treatment. In rare cases, trauma or torsion of epididymal cyst requires surgical exploration of the scrotum in order to make a clear diagnosis and timely treatment (2). EC torsion is extremely rare, and only 10 cases have been reported in the known literature (Table 1). As the second youngest EC torsion patient, the boy will become the eleventh case of EC torsion added to the literature (9).

**Table 1:** List of cases in literature.

Case no.	Authors and year	Age	Side(s)	Degree of torsion	History of scrotal trauma	Result of ultrasonic examination	Conservative treatment
1st	RI. Kaye et al. (3)	13-year-old	Left	360°	No	EC was found	No
2nd	N. Liolios et al. (4)	6-month-old	Left	360°	Not mentioned	Not done	No
3rd	E. Yılmaz et al. (5)	13-year-old	Left	720°	Yes	EC was found	No
4th	V Erikçi et al. (6)	11-year-old	Left	720°	No	EC was found	Yes
5th	Y. Akın et al., 2014	14-year-old	Bilateral	Not mentioned	No	ECs were found	No
6th	M. Ameli et al. (7)	14-year-old	Left	360°	Yes	EC was found	No
7th	C. Bleve et al. (2)	16-year-old	Right	360°	No	EC was found	No
8th	M. Messina et al. (8)	13-year-old	Left	Not mentioned	No	EC was found	Yes
9th	AMOM. Ozaal et al. (9)	8-year-old	Right	540°	No	EC was found	Yes
10th	M. Vafadar et al. (10)	11-year-old	Right	Not mentioned	No	EC was found	No
This case		3-year-old	Right	Not measured	No	EC was found	Yes

## Case report

**Figure 1:**
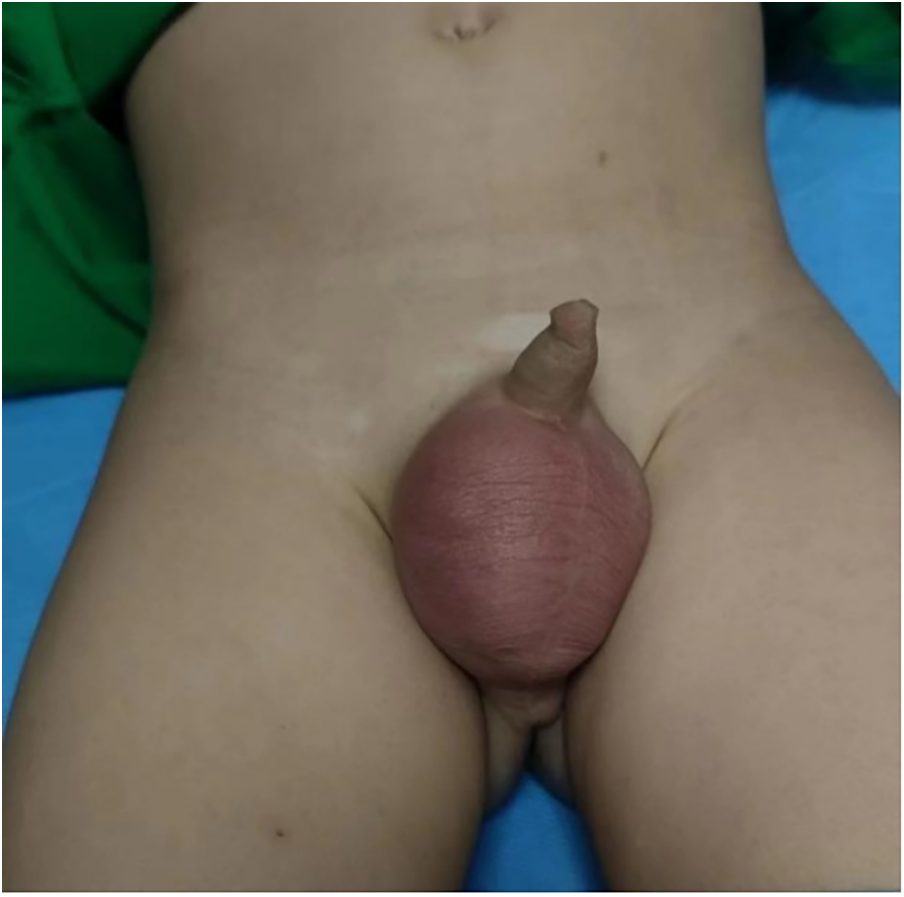
The red and swollen scrotum before operation.

**Figure 2:**
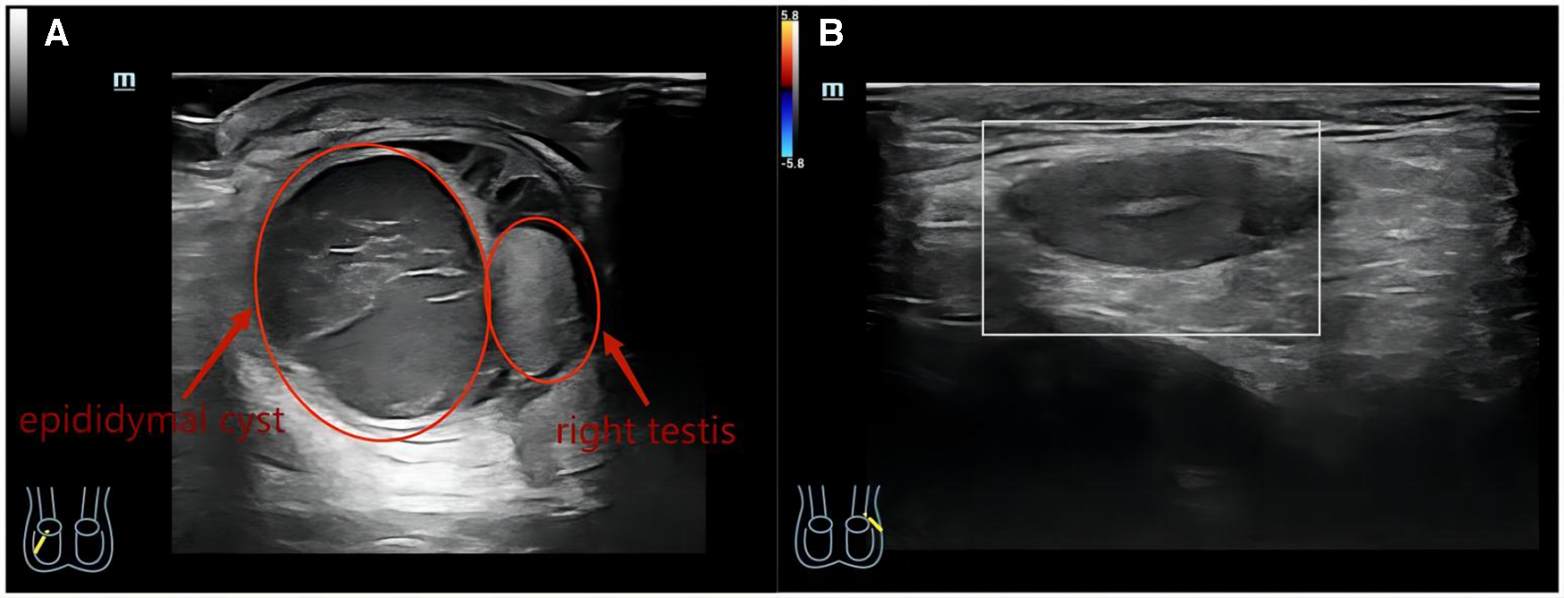
(**A**) Ultrasound image of right testis with epididymal cyst (on acute presentation). (**B**) Ultrasound image of the left testis in the left inguinal area.

**Figure 3:**
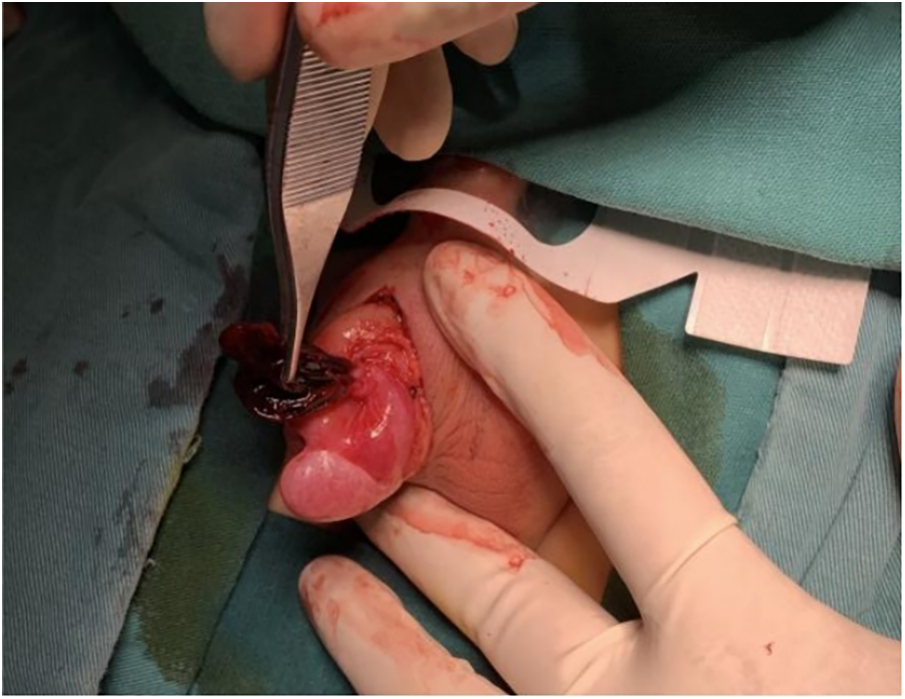
Normal right testis with twisted epididymal cyst.

**Figure 4:**
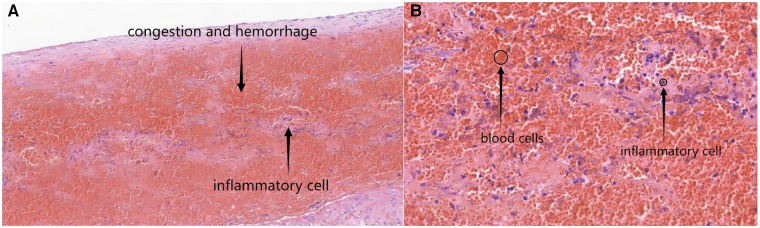
The microscopic views of the epididymal cyst characterized by congestion, hemorrhage and inflammatory cell [(**A**) HE,×100 and (**B**) HE,×400].

A 3-year-old boy came to our hospital because of redness and swelling of the scrotum for one day. His parents denied a history of scrotal trauma and the child had no fever, vomiting, or urinary symptoms. Physical examination showed redness and swelling of the bilateral scrotum (Figure 1), elevated skin temperature, especially on the right side, and positive tenderness. Ultrasonography immediately showed that the echo and perfusion of the right testis were normal and no space occupying was found. The cystic hypoechoic lesion in the right scrotum was 2.5 cm × 2.1 cm without perfusion and septum could be seen in it. The left scrotal was empty, and an 1.8 × 0.8 cm testicular echo was seen in the left inguinal area. Bilateral epididymal echo and perfusion were normal (Figure 2). The white blood cell count was 12.26 × 10^9^/L. As the boy's scrotal pain had not been relieved, and considering the high incidence of testicular torsion in scrotal emergencies and the possible impairment of testicular function, we decided to perform surgery immediately on the second day after the patient was admitted to the hospital. The patient underwent scrotal exploration under general anesthesia. After opening the edematous and thickened right scrotal membrane, we found a 2 × 2 cm dark red soft cystic mass on the upper pole of the right testis (Figure 3). The right testis and testicular appendages were normal and root torsion could be seen after fully dissociating the mass. The cyst was incised and decompressed, and dark red bloody fluid was seen flowing out. Relieve the torsion of the cyst, ligate and remove the cyst at the root, and then fix the right testis. After opening the left scrotum, we found normal left testis and testicular appendages in the left inguinal area, and performed left testicular descending fixation. Pathological examination showed fibrous tissue hyperplasia, extensive hyperemia with bleeding, and chronic inflammatory cell infiltration in the cyst (Figure 4). After operation, we continued to treat the patient with intravenous antibiotics and stopped when the patient was discharged from the hospital on the 4th day after operation. After a year of follow-up, the patient completely returned to normal.

## Discussion

EC usually occurs in adults around the age of 40. The incidence of EC in children is 5% Mur20%, and the incidence of EC increases with age (1). Currently, EC is widely regarded as an acquired disease, such as scrotal trauma, which is why EC usually occurs in middle-aged men. However, some studies suggest that they may be congenital abnormalities caused by hormone disorders in the process of embryonic growth and development (11). It has been reported that EC is associated with cryptorchidism and VHL. As we reported in this patient,it was complicated by cryptorchidism (1). Pain and scrotal mass are the most common clinical symptoms in patients with EC, and most patients seek medical treatment because of pain or scrotal swelling. But most patients with EC are asymptomatic, so they usually take conservative treatment. In up to 60% of patients, symptoms can disappear spontaneously, particularly when EC is less than 3 mm (1, 8, 9). However, once symptoms such as acute scrotal pain, swelling, and redness occur, it should be immediately identified in testicular torsion, testicular adnexal torsion, epididymitis and other scrotal emergencies. Because it is difficult to tolerate even short-term testicular ischemia, testicular torsion has become the most urgent disease to be identified or excluded in scrotal emergencies (12).

As reported by Schalamon et al., scrotal ultrasonography is capable of distinguishing surgical emergencies from other causes in 84% of children with acute scrotal pain (13). In our case, the patient presented with typical pain and scrotal swelling in a scrotal emergency. Scrotal ultrasonography showed that while the blood perfusion of the right testis was normal, a hypoechoic mass without blood perfusion was found in the right scrotum, and the patient had persistent scrotal pain. This forced us to explore the scrotum immediately.

This is the significance of our reporting on such cases. Because scrotal emergencies often occur in children, and whether the disease is related to the testicles and whether it will have an impact on the fertility in the future is always the most concern of all people. Therefore, if we can make the correct identification as soon as possible and carry out surgical exploration in time when necessary, it will provide a great guarantee for the quality of patients’ life in the future.

Our case is unique in that he is the second youngest case of EC torsion in the literature. Liolios et al. reported the youngest case (6 months old) of EC torsion (4). Moreover, this is also the fourth case of right EC torsion reported after Bleve et al. (2), Ozaal et al. (9) and Vafadar et al. (10).

All cases reported in the literature ultimately underwent surgical exploration. Although the cases reported by Messina et al. (8), Ozaal et al. (9), and Erikçi et al. (6) chose conservative treatment at first, they all underwent surgery to effectively relieve the scrotal emergency eventually.

## Conclusions

For patients with scrotal emergency, scrotal exploration should be carried out immediately if they have a recent history of scrotal trauma or are suspected of EC torsion. Otherwise, we should improve the ultrasound examination immediately and, if necessary, perform surgical exploration to clearly diagnose and deal with the disease.

## Data Availability

The original contributions presented in the study are included in the article/Supplementary Material, further inquiries can be directed to the corresponding author.
